# Supplementary material for: Development of Quantitative Real-Time PCR and Loop-Mediated Isothermal Amplification Assays for the Surveillance and Diagnosis of Herpes B Virus Infection
Source: Viruses. 2023 Oct 13;15(10):2086. doi: 10.3390/v15102086 (PMC10611326; doi:10.3390/v15102086)
Supplement: Supplementary file 1 [file viruses-15-02086-s001.zip › viruses-2652221-supplementary.pdf]

Supplemental Fugures and Table

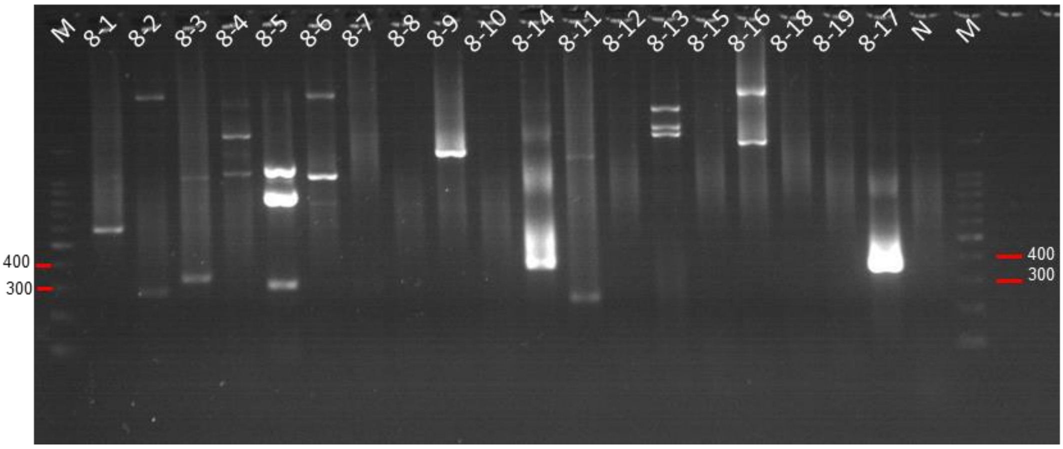

Figure S1. Nested PCR for gG-qPCR_positive samples
Nineteen samples (No.8-1, through 8-19) have been initially detected to be positive by gG-qPCR and further confirmed by nested PCR. The amplicon is 359 bp long. No. 8-1 through 8-19 have been loaded. M; 100 bp DNA ladder. Target bands are identified in No.8-14 and 8-17.

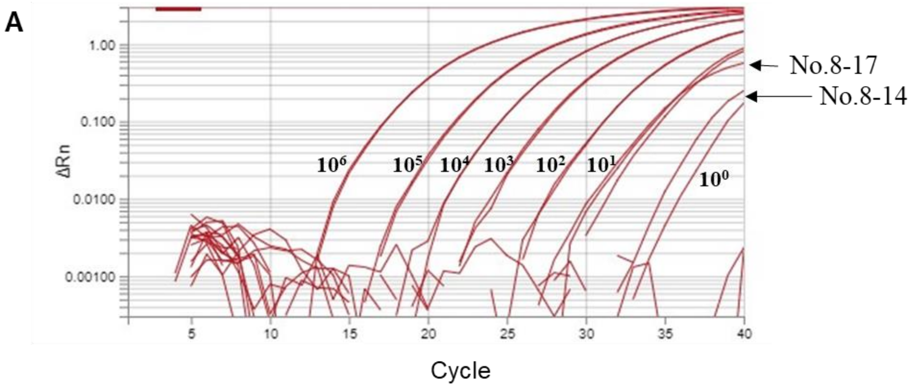

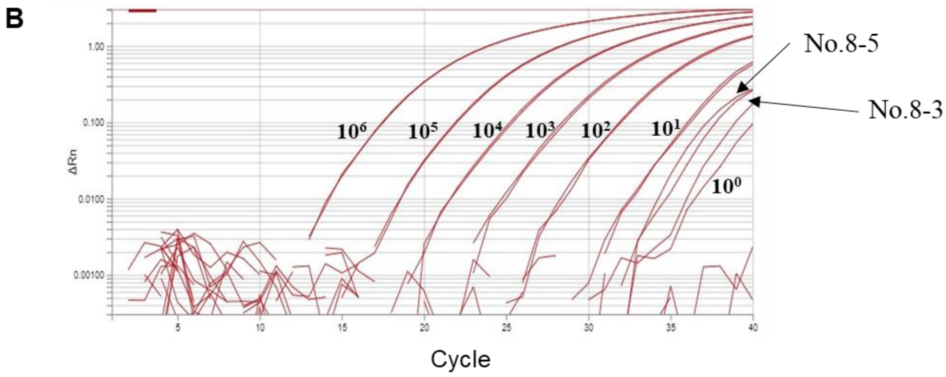

Figure S2. qPCR-gG amplification curve of No. 8-14 and No. 8-17 (A), and No. 8-3 and No. 8-5 (B).

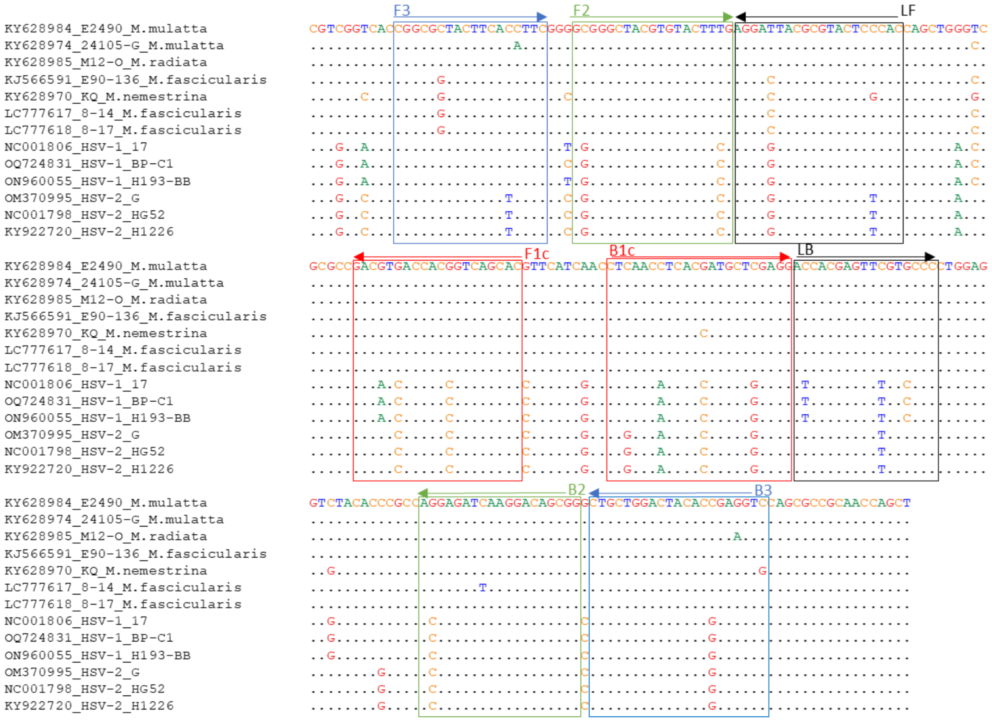

Figure S3. Sequence alignments of LAMP primer target regions of the genomes of BV and human herpesviruses (HSV-1 and HSV-2). The accession number of the genome sequence of the viruses are shown on the left.

Table S1. Sequences of the primers for conventional PCR and sequencing

Primer set	Primer	Sequence (5′-3′)
UL27-LAMP_1st	UL27-LAMP_Seq_F1	ACGTCATCATGCAGAACTCG
	UL27-LAMP_Seq_R1	GGTTGGAGAGGAAGGAGGAC
UL27-LAMP_nested	UL27-LAMP_Seq_F1	ACGTCATCATGCAGAACTCG
	UL27-LAMP_Seq_R2	GGCCCTCGAAGAAGGAGTAG
gG-qPCR_1st	gG-qPCR_Seq_F1	GACCCCGCGTACTGCTAC
	gG-qPCR_Seq_R1	CCCACCAGGATCTCGTAGTC
gG-qPCR_nested	gG-qPCR_Seq_F2	GCCGAYGTCGACAGACAT
	gG-qPCR_Seq_R1	CCCACCAGGATCTCGTAGTC
UL29-qPCR_1st	UL29-qPCR_Seq_F1	CAGTAGCGCAGGATCTGGTTG
	UL29-qPCR_Seq_R1	GCGTGATGGACCTCTTYAACA
UL29-qPCR_nested	UL29-qPCR_Seq_F2	CAGGATCTGGTTGGCCATGTAG
	UL29-qPCR_Seq_R1	GCGTGATGGACCTCTTYAACA
US5-6_1st	BV_US5-6_PCR_F1	CGTTTCCTCCCGTGGACTTC
	BV_US5-6_PCR_R1	GTCTGGAACGGGTTCTCCAC
US5-6_nested	BV_US5-6_PCR_F2	CTGACCCTGGCCGCCATG
	BV_US5-6_PCR_R2	GGTCCGTCTTCTGCTCCAGCG
US5-6_Seq	BV_US5-6_Seq_addF	TAAGTGGGAGGGGGATAAGG
	BV_US5-6_Seq_addR	CCGGCTTTTTAAAGGACAAA
